# The Design of Hyperbranched Polymer Biguanide Molecules with a Four-Arm Branched Core Structure Enhances Antibacterial Properties

**DOI:** 10.3390/polym16243481

**Published:** 2024-12-13

**Authors:** Bin Wang, Xu Meng

**Affiliations:** 1 Faculty of Quality Management and Inspection & Quarantine, Yibin University, Yibin 644000, China; binwang17711@163.com; 2 Chengdu Institute of Organic Chemistry, Chinese Academy of Sciences, Chengdu 610041, China; 3 School of Materials Science and Engineering, Changzhou University, Changzhou 213164, China

**Keywords:** hyperbranched polymer, polybiguanide, antimicrobial activity, antimicrobial mechanism

## Abstract

Amide–amine (PAMAM) dendrimers are biodegradable, non-immunogenic, genotoxic, and biocompatibible, which make them excellent materials for biological applications. In order to reduce the cytotoxicity of the designed branched molecules, a four-armed branched nucleus (B4) of PAMAM dendrimers as hyperbranched molecules was fused with polyhexamethylene biguanide (PHMB) (A2); hyperbranched polymeric biguanides (PAPBs) with a four-arm central core PAMAM structure were synthesized. The bactericidal and cell toxicity tests showed that PAPB had excellent bactericidal activity against both Gram-positive bacteria and Gram-negative, and the chemical binding of PHMB and PAMAM had synergistic effects. PAMAM reduced the toxicity of PAPB to 3T3 cells.

## 1. Introduction

A dendrimer is a kind of hyperbranched macromolecular compound with a definite three-dimensional structure. A complete dendrimer consists of a central core, several layers of repeated dendrimers, and a large number of peripheral groups [1]. The utility model has an adjustable inner cavity and a highly branched structure due to the easy modification of peripheral groups [2]. Compared with traditional linear analogs, the dendrimer has unique properties such as low viscosity, easy solubility, high reactivity, and high local group concentration due to its easy modification of peripheral groups, adjustable internal cavity, and highly branched structure [3,4,5]. Similarly to the dendrimer structure, a hyperbranched polymer is a kind of dendrimer with many end groups and a high degree of branching, which has the unique properties of dendrimers [6]. Hyperbranched polymers can usually be synthesized by a one-step method, so they are more suitable for industrial applications.

Because the peripheral functional groups of hyperbranched polymers can provide a higher local density, if these peripheral groups are modified into terminal groups with antimicrobial activity, the antibacterial properties of the modified hyperbranched polymer will be improved by increasing the concentration of local antimicrobial groups [7]. At the same time, the sequence of affinity between molecules and the bacterial plasma membrane is dendritic and hyperbranched polymeric antibacterial materials > conventional polymeric antibacterial materials > small molecular antibacterial materials [8]; therefore, the dendritic and hyperbranched polymeric antibacterial materials capture bacteria more easily. In summary, new hyperbranched antibacterial materials have great potential applications in industry and medicine compared with small-molecule antibacterial agents and linear polymeric antibacterial materials.

Tomalia et al. [9] have synthesized amide–amine (PAMAM) dendrimers, which have many advantages over other dendrimers. PAMAM dendronized polymers are nano-sized, spherical, monodisperse, highly branched polymers with well-defined structures and numerous primary amino groups on their peripheral surfaces [10]. Many studies have shown that PAMAM has good biocompatibility, high drug loading, biodegradability, non-immunogenicity, and genotoxicity. PAMAM complexes can be injected intravenously [11,12], and the holes in the PAMAM molecular structure and the large number of amino groups on its surface are conducive to the formation of stable complexes by electrostatic interaction with drugs [13], antibodies, nucleic acids [14], and fluorescent groups [15]. Therefore, it is widely used in different fields of biology and medicine and is a hotspot in the research of drug carriers.

When using antimicrobial agents, people often come into contact with them, so the biocompatibility of antimicrobial products and antimicrobial agents is one of the important factors affecting their application [16,17,18]. In order to enhance the antibacterial activity of antimicrobial agents, increase the variety of high-efficiency antimicrobial products, and reduce the cytotoxicity of antimicrobial agents, we designed and synthesized a class of novel low-toxicity hyperbranched polymer antimicrobial agents.

Because of their biodegradability and lack of immunogenicity and genotoxicity, as well as good biocompatibility, PAMAM dendrimer-based molecules are excellent materials for biological applications [19,20,21]. In this work, in order to reduce the cell toxicity of the designed branched molecule, we used a PAMAM dendrimer as the four-arm branched nucleus (B4) of the hyperbranched molecule and PHMB (A2) for melt polymerization to synthesize a hyperbranched polymer bisbiguanide PAPB with a four-arm central nucleus of a PAMAM structure. The molecular structure of the obtained hyperbranched products were characterized by Fourier transform infrared spectra (FTIR) and ^1^H NMR spectra. The antimicrobial properties were evaluated by the minimum inhibitory concentration (MIC) technique. Moreover, the antimicrobial mechanism was revealed by visualizing the bacteria morphology with SEM images.

## 2. Experimental

### 2.1. Materials

Methanol, methyl acrylate, ethylenediamine, trypsin, PBS buffer solution, and sodium dodecyl sulfonate were obtained from Chengdu Kelong Chemical Reagents Co., Ltd. (Chengdu, China). The preparation of polyhexamethylene biguanide (PHMB) was prepared based on our previous work [22]. Concentrated sulfuric acid, sodium chloride, trimesic acid, dicyandiamide (A.R.), and agar (gel strength > 1300 g/cm^2^, Hefei, China) were purchased from Sinopharm Chemical Reagent Co., Ltd. (Shanghai, China). Beef extract and peptone were obtained from Beijing Aoboxing Biotech Co., Ltd. (Beijing, China). Dimethyl sulfoxide (DMSO) and tetrazolium (MTT) were purchased from Sigma Chemical Reagent Co., Ltd. (St. Louis, MO, USA). All reagents were used as received without further purification.

Escherichia coli (*E. coli* ATCC25922) and Staphylococcus aureus (*S. aureus* ATCC209P) were kindly provided by the Institute of Sichuan Antibiotic Industry, State Pharmaceutical Administration of China. Mouse 3T3 fibroblasts were kindly provided by Sichuan University, Hwaseo School of Medicine.

### 2.2. Synthesis of Polyhexamethylene Biguanide Salt PAPB with Four-Arm Branched Nucleus

**Figure 1:**
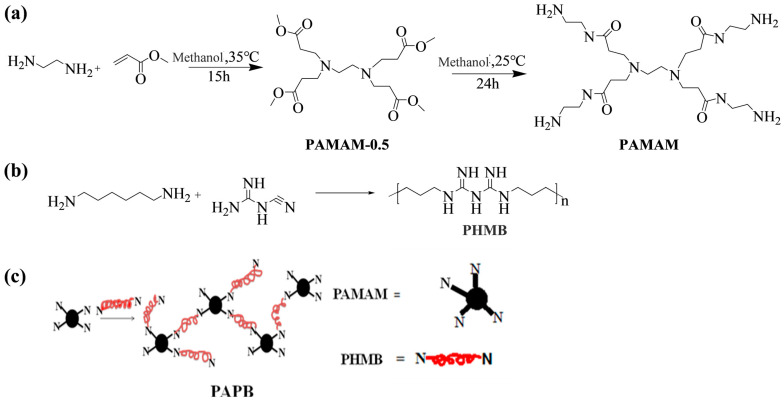
Synthesis equation of polyhexamethylene biguanide salt PAPB with four-arm branched nucleus. (**a**) Synthesis equation of PAMAM; (**b**) Synthesis equation of PHMB; (**c**) Synthesis equation of PAMAM.

Synthesis of PAMAM-0.5: The synthesis process is as shown in Figure 1a. In a round-bottom flask (250 mL), a solution of methanol (70 mL) and excess methyl acrylate (272 mmol) was mixed uniformly. The temperature of the system was reduced to about 5 °C, while the round-bottom flask was placed in an ice bath. Then, 30 mL of methanol solution containing 27.2 mmol of ethylenediamine was added slowly under a nitrogen atmosphere within 1 h. The reaction system was placed in an ice water bath for 30 min and then placed in a water bath at 35 °C for 15 h. The completion of the reaction was monitored by thin-layer chromatography (TLC). All the methanol and methyl acrylate were removed by vacuum distillation. PAMAM-0.5 was obtained with a yield of 98.5% (6.06 g). ^1^H NMR (300 MHz, CDCl_3_, ppm): δ 2.33 (t, *J* = 7.2, 8H), 2.38 (s, 4H), 2.65 (t, *J* = 7.2, 8H), 3.56 (s, 6H). IR (cm^−1^): 2952, 2828, 1738, 1437, 1329, 1198, 1174.

Synthesis of PAMAM: The synthesis process is as shown in Figure 1b. A methanol solution of a certain concentration of PAMAM-0.5 was added to the reaction vessel in a cold bath, and excess ethylenediamine (the molar ratio of ethylenediamine and PAMAM-0.5 is 24:1) was slowly added under a nitrogen atmosphere. After dripping, nitrogen was continued for 20 min. After sealing, it was placed in a water bath with a constant temperature at 25 °C for 24 h. The completion of the reaction was monitored by thin-layer chromatography (TLC). The methanol and excess ethylenediamine were removed by rotary evaporation. PAMAM was obtained with a yield of 99.8%. ^1^H NMR (300 MHz, CDCl_3_): δ 1.11 (s, 8H), 2.05 (t, *J* = 5.8 Hz, 8H), 2.14 (s, 4H), 2.36 (t, *J* = 5.8 Hz, 8H), 2.49 (t, *J* = 5.8 Hz, 8H), 2.96 (m, 8H), 7.75 (s). IR (cm^−1^): 3363, 3281, 2937, 2870, 1644, 1560, 1467, 1195, 1116.

Synthesis of PAPB: The synthesis process is as shown in Figure 1c. In a three-neck flask (250 mL) equipped with a mechanical stirrer, PAMAM and PHMB with different molar ratios were mechanically stirred under a nitrogen atmosphere to obtain a pale yellow lumpy solid by a melting reaction. ^1^H NMR (300 MHz, D_2_O, ppm): δ 1.30, 1.52, 2.38, 2.48, 2.64, 2.81, 2.88, 3.11, 3.24, 3.42, 3.53, 3.61. FT-IR (cm^−1^): 3342, 3199, 2938, 2866, 2150, 1655, 1560, 1466, 1357, 1295, 1249, 1188, 1115, 671, 605.

### 2.3. Characterizations

Structural characterization of synthetic products: The Fourier transform infrared spectra (FTIR, KBr) of polymers were recorded by a Nicolette Mx-1e Fourier transform infrared spectrometer. ^1^H NMR spectroscopy (AM 300 MHz NMR, Brooke, Billerica, MA, USA) was used to characterize their molecular structures.

Test for antibacterial properties: The qualitative and quantitative antibacterial activities of antibacterial drugs were proved by various techniques. The minimal inhibitory concentration (MIC) was determined by the broth double dilution method. To investigate the inhibitory effect of polymers on bacterial growth, *E. coli* and *S. aureus* were cultured on agar plates, and their fresh colonies were transferred to flasks with the help of a sterilizing ring. They were then incubated at 150 rpm for 18 h in a 37 °C incubator oscillator. The medium containing the required nutrients was used for culture. A total of 0.2 mL of bacterial suspension was transferred into each test tube, and 1.8 mL of different concentrations of antibacterial agents were added in triplicate before inoculation, from 1000 to 0.1 μg·mL ^−1^ (different test concentrations were 1000, 100, 10, 1, and 0.1 μg·mL^−1^, respectively). A total of 0.2 mL bacterial suspension was added to 1.8 mL double-distilled water as a blank sample, and the OD of bacteria was determined at 700 nm. All the tubes were placed in an incubator oscillator for 24 h at a temperature of 37 °C and were oscillated at a rate of 150 rpm. There was a good linear relationship between bacterial biomass and optical density at 700 nm. Thus, bacterial counts in the control and treatment groups were monitored with a U-2010 spectrometer (Hitachi, Tokyo, Japan) by measuring optical density at 700 nm. The results are expressed as the percent inhibition of bacterial growth. The inhibition percentage is calculated according to the following formula, named Equation (1):

(1) $$\mathrm{Inhibition}\;\mathrm{percent}=\frac{{\mathrm{OD}}_{700}\;\mathrm{control}\;-{\;\mathrm{OD}}_{700}\;\mathrm{assay}}{{\mathrm{OD}}_{700}\;\mathrm{control}}\times 100\%$$

Test for cytotoxicity properties: Mouse 3T3 fibroblasts were passaged at least twice and digested with 0.05% trypsin. Log cells were collected and the cell suspension concentration was adjusted. Then, it was added to 96-well plates, with 200 mL per well and 4000–10,000 cells per well. The 96-well plates were cultured in a 37 °C 5% CO_2_ incubator for 12–24 h. The plates were randomly divided into a negative control group and different antimicrobial treatment groups. The blank control group was treated with 200 μL PBS buffer, and the treatment group was treated with 200 mL antimicrobial agent. The final concentrations were 1000 μg/mL, 100 μg/mL, 10 μg/mL, 1 μg/mL, and 0.1 μg/mL, respectively, and the groups were cultured for 24 h in 80 mL fresh medium and 20 μL MTT (0.5%) solution for 4 h. After centrifugation, 150 μL of MSO was added into each well and 100 μL of acidified sodium dodecyl sulfonate was added directly (pH = 4.7). Absorbance values (OD values) of the wells were measured at 570 nm, while background wells (medium, MTT, DMSO) and control wells (same concentration of drug medium, medium, MTT, DMSO) were set. Then the absorbance (OD) was measured using an Elisa (DeTie, Nanjing, China). The cell relative survival is calculated according to the following formula, named Equation (2):

(2) $$RSR(\%)=\frac{\mathrm{OD}\;\mathrm{value}\;\mathrm{of}\;\mathrm{test}\;\mathrm{group}}{\mathrm{OD}\;\mathrm{value}\;\mathrm{of}\;\mathrm{control}\;\mathrm{group}}$$

Analysis of surface morphology by SEM: After centrifugation at 3000 rpm for 3 min, *E. coli* cells were washed twice with PBS and redispersed with PBS solution. Different concentrations of polymers were added into the suspension of *E. coli* and shaken for 30 min. A glutaraldehyde solution (2.5%) was added to the untreated *E. coli*, and the surface morphology of the bacterial cells was fixed in the treated *E. coli* suspension. The *E. coli* suspension was then centrifuged at 3000 rpm for 3 min, washed three times with PBS, and re-dispersed in PBS. *E. coli* cells were dehydrated by gradient series ethanol (50, 70, 80, 90, and 100%, respectively) for 15 min and then air-dried in a vacuum dryer. Scanning electron microscope images were observed by SEM (Leitz AMR-1000, Oberkochen, Germany).

## 3. Results and Discussion

### 3.1. Synthesis and Characterization of Hyperbranched Polymerized Biguanides with PAMAM Structure

The synthesis of PAMAM had two steps. Firstly, the semi-algebraic PAMAM was obtained by a Michael addition of ethylenediamine and methyl acrylate. Then, the semi-algebraic PAMAM was used to react with excess ethylenediamine for the amidation of ester groups to obtain the whole algebraic dendrimer. The yields of both steps were very high. Under the best reaction conditions, the yield could reach 98–100%, which was almost quantitative polymerization.

**Figure 2:**
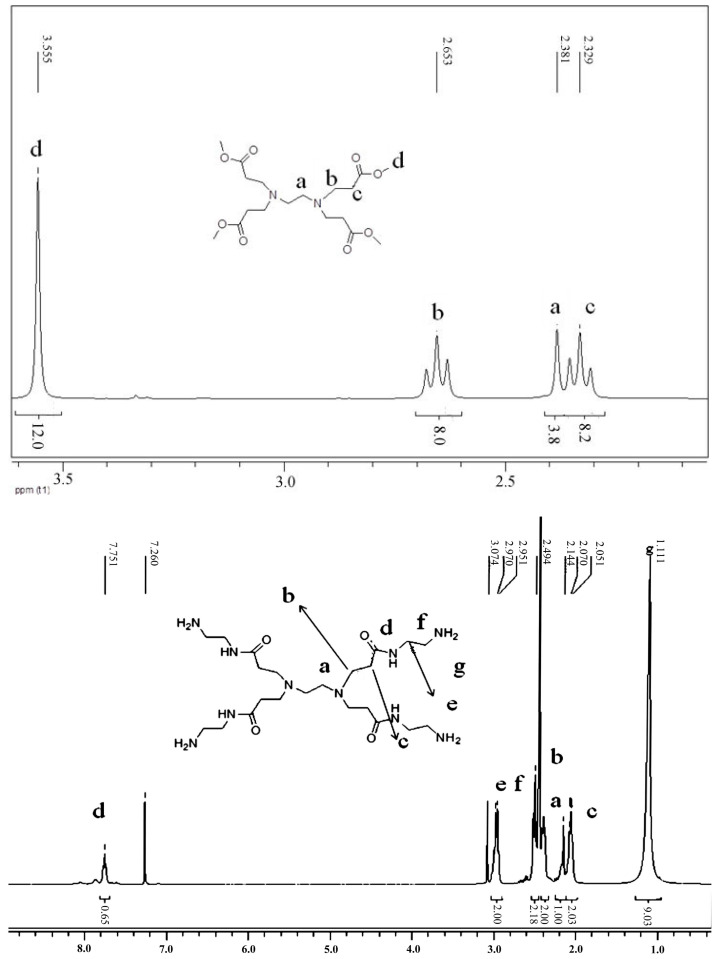
^1^H-NMR spectra of PAMAM-0.5 and PAMAM.

Figure 2 shows the ^1^H-NMR spectra of PAMAM-0.5 and PAMAM. ^1^H-NMR spectra of PAMAM-0.5 showed the singlet at 3.56 ppm, belonging to oxymethyl (OMe, d). The triplet at 2.65 ppm was observed and assigned to carbonyl methyl. The chemical shift in the methylene group moved to a low field due to the factor of electron absorption of the carbonyl group. The singlet at 2.38 ppm belonged to CH_2_ (CH_2_, a), and the triplet at 2.33 ppm was observed and assigned to CH_2_ (CH_2_, c). The integration ratio of the four types of hydrogen was CH_2_ (CH_2_, a):(C=OCH_2_, b):CH_2_ (CH_2_, c):(OMe, d) = 4:8:8:12, which corresponded to the ratio of the number of different types of hydrogen in the molecular structure, proving the successful synthesis of PAMAM-0.5.

^1^H-NMR spectra of PAMAM showed the peak at 7.75 ppm, belonging to hydrogen (C(=O)NH, d) in the imide group. Since it belonged to active hydrogen, the hydrogen integral of it was less than the actual amount of hydrogen. δ = 1.11 was the chemical shift value of NH_2_ (g). The peak corresponding to the hydrogen atom in -OCH_3_ at 0.56 ppm (CH_3_, d in ^1^H-NMR spectra of PAMAM-0.5) completely disappeared, indicating that all the ester groups of the 0.5 generation product were converted to amide groups. The ^1^H-NMR spectra of other methylene groups were as follows: δ = 3.46 (CH_2_, e), δ = 2.91 (CH_2_, f), δ = 2.64 (CH_2_, b), δ = 2.46 (CH_2_, a), and δ = 2.28 (CH_2_, c).

**Figure 3:**
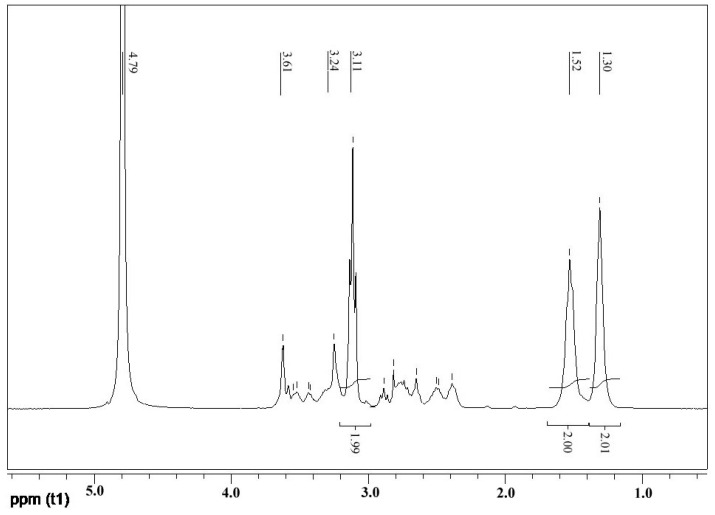
^1^H-NMR spectrum of PAPB1.

A2 (PHMB) and B4 (PAMAM) monomers were used to synthesize the hyperbranched polymer PAPB by melt polymerization, and the structure of the final product was determined by FT-IR and NMR. Figure 3 shows the ^1^H-NMR spectra (D_2_O) of PAPB1. Because of the use of deuterated water as a nuclear magnetic solvent, the active hydrogen on the amino and imino groups in the PAPB1 molecule is rapidly exchanged with the deuterated reagent, and their peaks disappear. It can be seen from the figure that δ = 1.30, δ = 1.52, and δ = 3.11 were the three kinds of hydrogen of hexamethylenediamine of PAPB1, and the integral ratio of hydrogen was 1:1:1. When PHMB reacted with PAMAM, some of the amine groups were replaced by biguanides, and that was why the ethyl hydrogen connected to it shifted to the lower field (δ = 3.61, δ = 3.53). The peak positions of the rest of the proton hydrogen were consistent with PAMAM.

**Figure 4:**
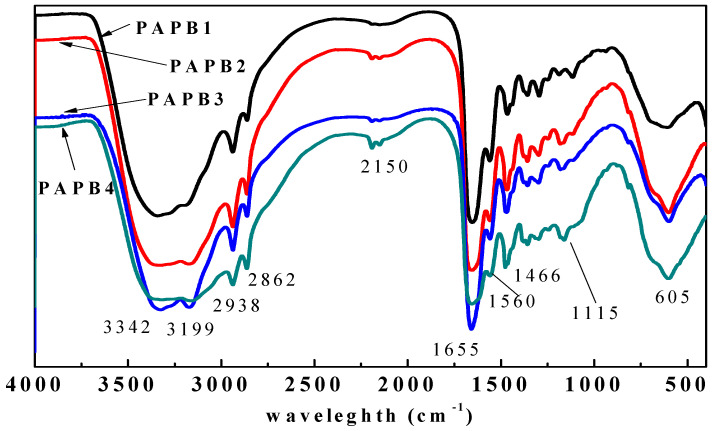
The FT-IR spectrum of PAPB.

The FT-IR spectrum of the PAPB is shown in Figure 4. The 3342 cm^−1^ and 3199 cm^−1^ peaks were assigned to the stretching vibration of -NH_2_. The asymmetric stretching vibration (ν_as_) and symmetric stretching vibration (ν_s_) of -CH_2_- were observed at 2938 cm^−1^ and 2866 cm^−1^, respectively. The characteristic bands of amide bonds (ν_C=O_) and (δ_NH_ and ν_CN_) were noticed at 1655 cm^−1^ and 1560 cm^−1^, respectively. The bending vibration (δ_as_) of -CH_2_- was observed at 1466 cm^−1^. The 1188 cm^−1^ and 1115 cm^−1^ peaks were assigned to the stretching vibration (ν_CN_) of primary and tertiary amines, respectively. It was shown that the PAPB1 contained characteristic groups such as -NH_2_, -CH_2_-, -CONH-, and C = N, which were consistent with the characteristics of the theoretical structure.

### 3.2. Antibacterial Property

Table 1 shows the antibacterial results of PHMB, PAMAM, and PAPB against *E. coli*. It can be seen from the results of the inhibition zone that the inhibition zone diameter of the branched modified product PAPB was larger than that of PHMB and PAMAM, indicating that the chemical combination of PHMB and PAMAM could improve the antibacterial performance of antibacterial agents. The results of the antibacterial rate were consistent with this result, with PAPB (98.6~99.0%) > PHMB (96.8%) > PAMAM (92.4%). When the content of PHMB in the product increased, the inhibition zone diameter decreased from 5.9 cm (PAPB1) to 3.6 cm (PAPB4). Considering the small change in the bacteriostatic rate of PAPB, it may be due to the increase in PHMB structure content in the PAPB molecule, which makes the interaction between the PAPB molecule and agar medium increase and the diameter of the bacteriostatic circle decrease [23]. The experimental results of MIC showed that the MIC of the product PAPB increased with the increase in the content of PHMB, PAPB4 = PAPB3 > PAPB2 > PAPB1. This might be that the increase in the content of the PHMB structure in the periphery of the branching center core, which caused the encapsulation of effective bactericidal active groups, rendered a decrease in antibacterial capacity. However, the overall antibacterial effect was still stronger than that of PHMB and PAMAM.

**Figure 5:**
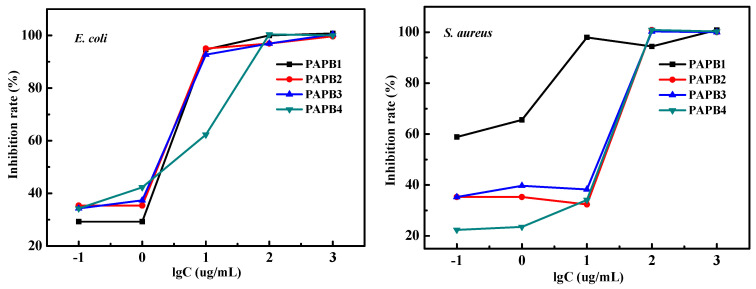
Inhibition rate of PAPB with various concentrations against *E. coli* and *S. aureus*.

Figure 5 shows the inhibition rate of PAPB with various concentrations against *E. coli* and *S. aureus*. As can be seen from the figure, the bacteriostatic rate of PAPB showed a concentration-dependent relationship. PAPB with a low concentration had a low bacteriostatic rate, and when a certain concentration was reached, the bacteriostatic rate increased rapidly to more than 90%. For *E. coli*, the bacteriostatic rate was 30–40% when the concentration was less than 1 μg·L^−1^, and the bacteriostatic rate was more than 90% when the concentration was 10 μg·L^−1^. For *S. aureus*, PAPB1 had the best antibacterial performance. The antibacterial rate could reach 60% when the concentration of PAPB1 was 0.1 μg·L^−1^. In summary, PAPB had excellent antimicrobial activity against both *E. coli* and *S. aureus*.

**Figure 6:**
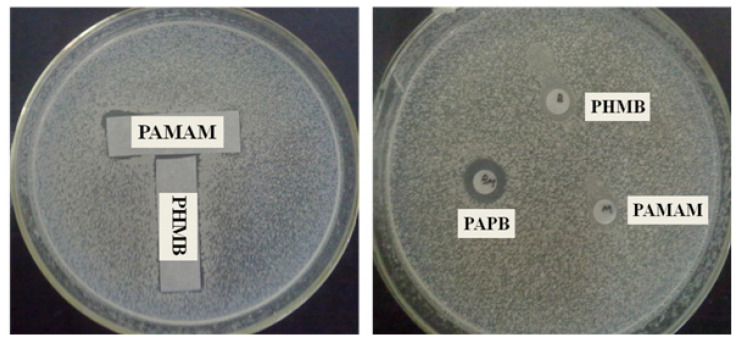
Combined test and inhibition zone test of PHMB and PAMAM against *E. coli*.

Figure 6 shows the antibacterial effect of the combination of PAMAM and PHMB and the inhibition zone of PHMB, PAMAM, and PAPB. It can be seen from the results of the combined tests that the bacteriostatic region between PHMB and PAMAM did not change, which indicates that when the two were physically mixed, they showed an irrelevant effect. Combined with the experimental results of the inhibition zone, the order of antibacterial performance was PAPB > PHMB and PAMAM, indicating that the enhanced effect of branched modified biguanides on sterilization was due to the chemical bond between PHMB and PAMAM.

**Figure 7:**
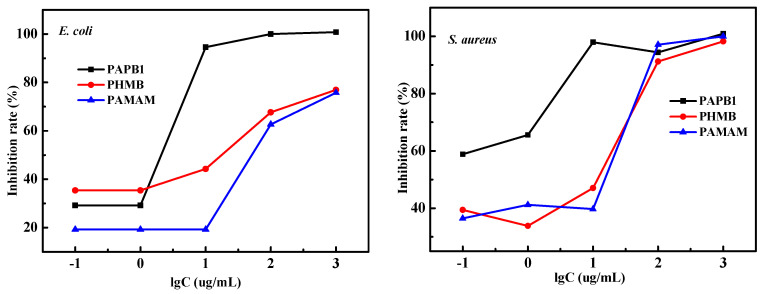
Inhibition rate of PAPB1, PHMB, and PAMAM with varied concentrations against *E. coli* and *S. aureus*.

In order to quantify this chemical binding synergy, the inhibition rates of PHMB, PAMAM, and PAPB against *E. coli* and *S. aureus* were determined by turbidimetry. Figure 7 shows the experimental results of the inhibition rate.

According to the inhibition rates of PAPB1, PHMB, and PAMAM against *E. coli* and *S. aureus*, the inhibition rate effect of PAPB1 could reach more than 90% against Gram-negative bacteria and Gram-positive bacteria with a concentration of 10 μg·L^−1^, while the inhibition rate effect of PHMB and PAMAM against Gram-negative bacteria was greater than that against Gram-positive bacteria, indicating that the antibacterial spectrum of PAPB1 was wider. At the same time, the order of inhibition rate was PAPB1 > PHMB > PAMAM, which further illustrated that the chemical binding products of PHMB and PAMAM had a synergistic antibacterial effect.

Compared to polyhexamethylene biguanide functionalized hyperbranched DPB1 [22], as shown in Table 1, PAPB1 exhibited superior performance in antibacterial activity such as the inhibition zones, MIC, and the antibacterial rate. This indicates that the branched structure improvement of PHMB with a four-branch central core (PAMAM) was superior to that with a three-branch central core (DT).

### 3.3. Analysis of Antibacterial Mechanism

**Figure 8:**
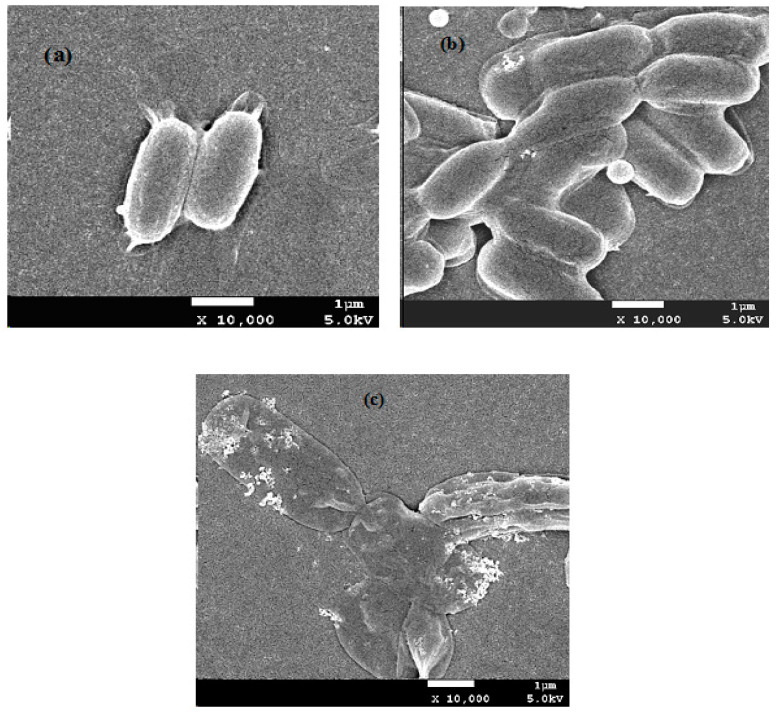
SEM images of the *E. coli* and PAPB1-treated *E. coli*. (**a**) Untreated *E. coli*; (**b**) PAPB1 (20 ppm, 30 min)-treated *E. coli*; (**c**) PAPB1 (200 ppm, 30 min)-treated *E. coli*.

SEM images were used to show the morphological changes in PAPB1-treated *E. coli*, revealing the antibacterial mechanism of the antibacterial agent. Figure 8 shows the SEM images of *E. coli* before and after treatment with different concentrations of PAPB1. As can be seen from Figure 8b, after treatment with a low concentration of PAPB1 (20 ppm) for 30 min, the full and smooth cell membrane surface of *E. coli* appeared with a slight sag morphology and peeling. That is, under a low concentration of antibacterial agent, the cell membrane was less affected by interference. Although it showed a slight depression of the cell membrane, most bacterial cell membranes still maintained a relatively complete structure and the bacteria profile was relatively clear, which maybe died because PAPB had adsorbed onto their cell surfaces had not disrupted the cell membranes. When the concentration of the antibacterial agent increased to 200 ppm, the cell membrane of *E. coli* ruptured and shrank, which caused a large amount of internal soluble matter to leak. This conclusion was consistent with the antibacterial mechanism of a guanidine polymer [24].

### 3.4. Toxic Effect of PAPB on 3T3 Cell

**Figure 9:**
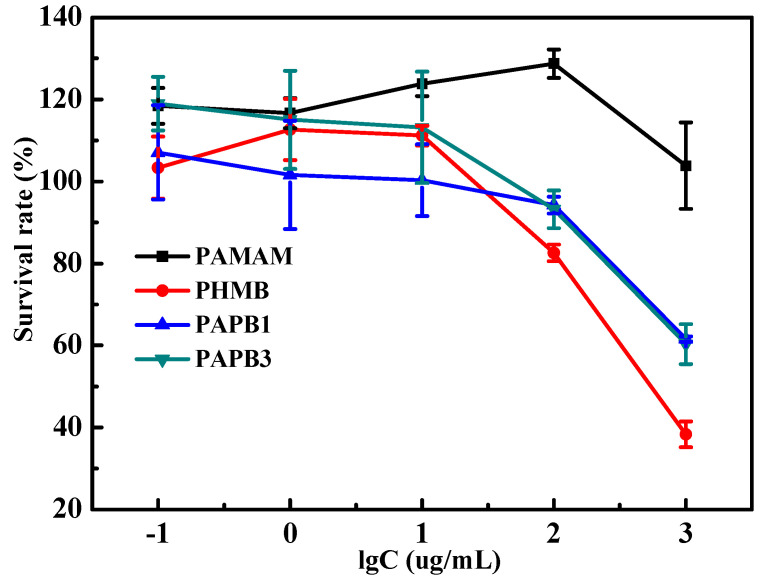
Toxicity of PAMAM, PHMB, and PAPB on 3T3 cells with varied concentrations.

Figure 9 shows the toxicity of PAMAM, PHMB, and PAPB on a 3T3 cell, which is expressed by the survival rate of the 3T3 cell. It can be seen from the figure that the cell survival rates were dependent on the concentration of antibacterial agent. With the change in the concentration, all the cell survival rates showed a certain change. When the concentration of the antibacterial agent was below 100 micrograms per liter, the survival rate of 3T3 cells often exceeded 100%. This could be due to experimental errors, or it could indicate that PAMAM has a certain proliferative effect on 3T3 cells. Either way, it suggests that PAMAM exhibits good biocompatibility at low concentrations. With the increase in the concentration, the cell survival rate decreased slightly. And when the concentration of the antibacterial agent exceeded 100 μg·L^−1^, the survival rate of 3T3 cell varied greatly. The survival rate of PAMAM-treated 3T3 cells was still greater than 100%, which proved that PAMAM is indeed a class of compounds with biocompatible and low-toxicity properties [11]. The effect of antibacterial agents on the survival rate of 3T3 cells was PAMAM-treated 3T3 cells > that of PAPB1-treated 3T3 cells ≈ that of PAPB3-treated 3T3 cells > that of PHMB-treated 3T3 cells. The survival rate of PHMB-treated 3T3 cells with 1000 μg·L^−1^ was 38%, while that of PAPB-treated 3T3 cells was 60%, which indicates that the introduction of PAMAM with less biotoxicity into PHMB was beneficial to reduce the molecular toxicity.

## 4. Conclusions

A hyperbranched polyhexamethylene biguanide (PAPB) derivative with a four-arm branched nucleus (PAMAM) was designed and synthesized successfully. PAPB has excellent bactericidal activity against both Gram-positive bacteria and Gram-negative bacteria, and the chemical binding of PHMB and PAMAM has antibacterial synergy. The antibacterial mechanism of PAPB showed that, similar to cationic polymers, the target of PAPB is the bacterial cell membrane, which reduces membrane fluidity and enhances membrane permeability, and a large amount of intracellular lysate leakage caused bacterial cell death. Hyperbranched polyhexamethylene biguanide PAPB with PAMAM-branched nuclei had a reduced toxic effect on 3T3 cells compared with PHMB, suggesting that the introduction of PAMAM favors the reduction in the toxicity of polyhexamethylene biguanide on 3T3 cells.

## Figures and Tables

**Table 1:** Antibacterial activity of PHMB, PAMAM, and PAPB against *E. coli*.

	PHMB	PAMAM	PAPB1	PAPB2	PAPB3	PAPB4	DPB1 [22]
Inhibition zone (cm)	4	1	5.9	5	4	3.6	6.8
MIC (ppm)	128	256	32	64	128	128	64
Antibacterial rate (%)	96.8	92.4	98.6	99.0	98.9	98.7	70.4

## Data Availability

The original contributions presented in this study are included in the article. Further inquiries can be directed to the corresponding author.
